# Anti-Neutrophil Cytoplasmic Antibodies: To Care or Not to Care

**DOI:** 10.7759/cureus.17094

**Published:** 2021-08-11

**Authors:** Sara Shahid, Syed H Alam, Lillian Gaber, Salman Ahmed

**Affiliations:** 1 Medicine, Lahore Medical And Dental College, Lahore, PAK; 2 Rheumatology, Houston Methodist West Hospital, Houston, USA; 3 Pathology, Houston Methodist Hospital, Houston, USA; 4 Internal Medicine, Baylor College of Medicine, Houston, USA

**Keywords:** anti-neutrophil cytoplasmic antibody associated vasculitis, anca, crescentic glomerulonephritis, renal vasculitis, ana, mpo antibody, pr3 antibody, acute kidney injury, aki, cyclophosphamide

## Abstract

Anti-neutrophil cytoplasmic antibodies (ANCA) associated vasculitis is a disease process with a wide range of presentations, from asymptomatic or minimally symptomatic disease with positive laboratory testing, to florid acute end-organ damage. Consensus has not been established as to the frequency and/or protocol by which ANCA testing should be repeated. We present the case of a 53-year-old woman who initially came to medical attention with persistent dyspnea and pulmonary infiltrates presumed to be due to acute exacerbation of chronic diastolic congestive heart failure. Extensive infectious disease testing was negative, but ANCA testing was positive. However, because antinuclear antibody (ANA) interference in the original sample rendered the test result difficult to interpret, the test was not repeated. The patient presented eight months after the initial hospitalization with acute hypoxemic respiratory failure requiring intubation, with an ANCA titer of 1:1280 with a negative ANA titer, and renal biopsy-proven severe crescentic glomerulonephritis. In the discussion of our case, we review the importance of interpreting ANCA testing in the correct clinical context. The ANCA laboratory testing requires cautious interpretation, and diagnosed ANCA-associated vasculitis (AAV) requires vigilance for prompt and proactive treatment.

## Introduction

Anti-neutrophil cytoplasmic antibody (ANCA) associated vasculitis is a disease process with a wide range of presentations. Complicating the diagnosis of ANCA associated vasculitis (AAV) is the interpretation of laboratory test results for this disease process. Patients may be found incidentally positive for ANCA, may be found to have ANCA in the setting of minimal complaints such as arthralgias or a minimally bothersome cough, or may be found to have ANCA in the setting of a dramatic clinical presentation such as new-onset acute renal failure or acute hypoxemic respiratory failure secondary to diffuse alveolar hemorrhage. The astute clinician and high-quality health system must maintain vigilance to answer one question: What is to be done with a positive ANCA test result, in any given context?

Patients with positive ANCA can have a wide spectrum of diseases, and some of them may be non-vasculitides as well. The latter is due to the false positivity of ANCA. The age, clinical symptoms and signs, and the disease epidemiology differ based on the condition which resulted in ANCA positivity.

## Case presentation

Our patient was a 53-year-old woman who presented with the chief complaint of dyspnea. Her only medical condition at the time of index hospitalization was morbid obesity and well-controlled hypothyroidism. She described dyspnea of moderate intensity, which had been progressively worsening for one week prior to her admission. She did not report cough, epistaxis, arthralgia, chest pain, angina, or claudication. The patient’s initial vital signs on presentation were temperature of 99.8 degrees Fahrenheit, pulse 98 beats per minute, blood pressure 145/67 mmHg, respiratory rate 22 breaths per minute, and oxygenation was 100% on room air. The examination was notable for morbid obesity, as well as for tachypnea appreciated on pulmonary examination. Initial biochemical testing revealed blood urea nitrogen (BUN) of 11 mg/dL, serum creatinine (SCr) of 0.87 mg/dL, and estimated glomerular filtration rate (eGFR) of 86 mL/min. A computed tomographic angiogram (CTA) of the chest with pulmonary embolism protocol revealed pulmonary interstitial infiltrates suggestive of pulmonary edema and was negative for pulmonary embolism. She was admitted to the inpatient unit and placed on diuretics and supplemental oxygen for management of a presumptive diagnosis of acute exacerbation of chronic diastolic congestive heart failure. Empiric antibiotics, vancomycin and piperacillin-tazobactam, were started in the setting of possible concomitant pneumonia.

The pulmonary infiltrates persisted despite diuresis and antibiotics. For this reason, the infectious diseases service was consulted. The patient underwent extensive testing for infectious as well as non-infectious causes of persistent pulmonary infiltrates. Polymerase chain reaction testing for COVID-19, several other coronaviruses, adenovirus, influenza virus, parainfluenza virus, human metapneumovirus, respiratory syncytial virus, enterovirus, rhinovirus, Bordetella, chlamydia, mycoplasma pneumoniae, and methicillin-resistant staphylococcus aureus all resulted negative. HIV antibody test was negative. An ANCA test was performed and returned with a positive result, reported as: “P-ANCA positive. The titer of P-ANCA cannot be reliably determined due to interference from positive ANA staining at a titer of 1:20 on IFA slides.” Because of this factor affecting testing, no ANCA titer was reported. Myeloperoxidase (MPO) antibodies were categorized as “moderate to strong positive” with titer >30 units, while proteinase-3 (PR-3) antibodies were negative. The patient’s dyspnea improved and she was discharged from the hospital. The patient’s ANCA test was not repeated.

Some eight months after the index hospitalization, the patient presented to the hospital in extremis. She was severely dyspneic and had evidence of pulmonary and peripheral edema. Laboratory testing on presentation revealed BUN of 176 mg/dL and SCr of 19.38 mg/dL. Potassium was 6.2 meq/L. An indwelling urinary catheter was placed. She was noted to be oliguric. The patient was intubated within six hours of presentation. Dialysis was initiated and continued once daily for three days. Testing for ANCA revealed P-ANCA positivity with a titer of 1:1280. Once again, MPO antibodies were categorized as “moderate to strong positive” with titer >30 units, while proteinase-3 (PR-3) antibodies were negative.

**Figure 1:**
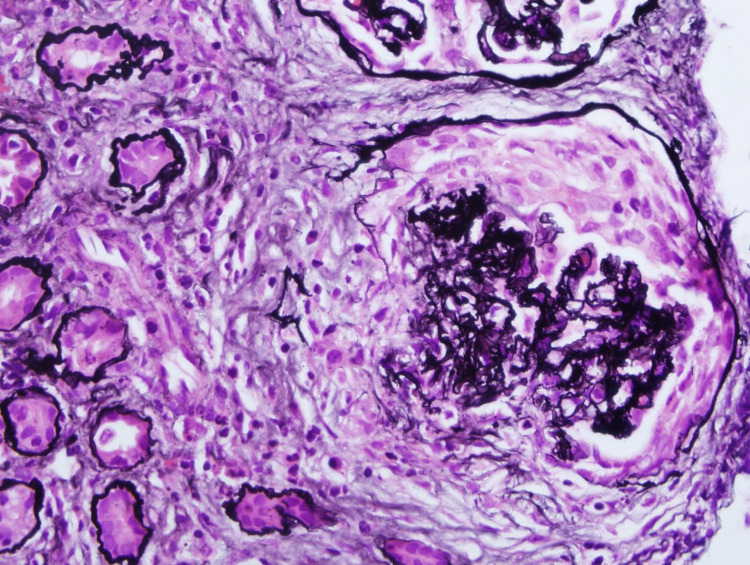
Epithelial crescent filling the urinary space and eroding Bowman’s capsule. Glomerular tuft enclosed by the crescent shows sclerosis, loss of capillary spaces, and near complete loss of cells [Jones stain, 40X initial magnification].

**Figure 2:**
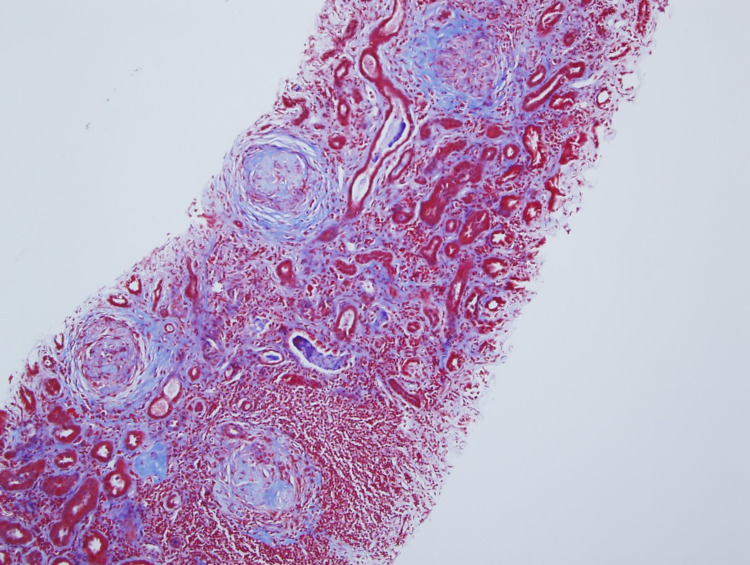
Trichrome stain highlighting interstitial fibrosis. Glomerular crescents are in the fibrous sclerosing phase [trichrome stain, 10X initial magnification].

A renal biopsy was performed that demonstrated active crescent formation on a background of many glomeruli that were already globally sclerosed (Figures 1-2). The patient was treated with pulse dose steroids, IV cyclophosphamide, and seven sessions of plasmapheresis. She did not recover sufficient renal function by the time of hospital discharge to be able to discontinue dialysis. After six out of seven total sessions of plasmapheresis, the ANCA titer had been reduced to 1:80. She continued outpatient dialysis. A 24-hour urine collection performed approximately one month after completion of plasmapheresis revealed a creatinine clearance of approximately 5 mL/min. After approximately three months of dialysis under the diagnosis of acute kidney injury, the patient was declared to have transitioned into end stage renal disease (ESRD).

## Discussion

Antineutrophil cytoplasmic antibodies (ANCA) are directed against different components of neutrophils. To observe the antibodies, the neutrophils are fixed with ethanol, and the subsequent patterns are observed. The pattern observation and analysis is dependent on the observer, and inter-observer variability has been reported [1]. Initial studies on ANCA and their isolation, however, were done in research laboratories, hence it is recommended that these studies are analyzed at an experienced laboratory. In the case of our patient, the ANCA test was read at an experienced tertiary care hospital’s laboratory.

The two main patterns of ANCA are cytoplasmic or C-ANCA, and perinuclear or P-ANCA. In the case of C-ANCA, the antibody staining is throughout the cytoplasm, and conversely, with P-ANCA, the staining is limited to the perinuclear region [2]. The identification of ANCA is done by indirect immunofluorescence (IF). There are multiple types of ANCA patterns, however, it is important to determine the specific antigen targeted of these antibodies. This has also led to a shift in clinical practice, where the division of vasculitis is being considered based on the antigenic target, rather than viewing the diseases by their eponyms [3].

Stone et al. have shown in their study that the sensitivity of IF is higher than enzyme-linked immunosorbent assay (ELISA) (67% vs 55%), however, the specificity of ELISA is higher, 99% vs 93% [4]. The two main antigens to consider are proteinase 3 (PR3) and myeloperoxidase (MPO). PR3 is a 29-kd serine protease, and it is housed in the azurophilic granules of neutrophils and peroxidase-positive lysosomes of monocytes [5]. MPO is found in the same cellular compartments, but its weight is 140-kd. The identification of the antigens is done by ELISA. In most cases, C-ANCA targets the PR3 antigen, and the P-ANCA targets the MPO antigen [6-7]. However, this association between the antibodies and antigens has exceptions. PR3 can infrequently be responsible for P-ANCA antibodies, and MPO can sometimes cause a C-ANCA pattern. Atypical ANCA antibodies patterns can be seen with other conditions with different antigenic targets, and this antibody pattern is observed to be perinuclear, however, testing for the specific antigenic target can help in this situation. Due to the presence of “atypical” P-ANCA, the P-ANCA has lower specificity for vasculitis than C-ANCA.

Of note, there are no universally acceptable “reference ranges” for ANCA. For our patient, her P-ANCA was at a titer of 1:1280, which would generally denote a very high titer. Another problem to consider with ANCA is that the pattern of P-ANCA can be difficult to distinguish from the pattern caused by ANA antibodies. Hence, checking for ANA in cases of P-ANCA positivity is prudent to avoid false-positives. In our patient, on the second hospital admission, the ANA was negative which increases the specificity of the P-ANCA manifold. Also, the most important information in the case for our patient was that the MPO antibody was positive. This is important since the P-ANCA on its own has lower specificity and higher false-positives than C-ANCA.

There are certain disease associations with each pattern of ANCA, and there is a great amount of historic importance to this. When considering ANCA-positive vasculitis, generally, we refer to granulomatosis with polyangiitis (GPA), microscopic polyangiitis (MPA), and eosinophilic granulomatosis with polyangiitis (EGPA) [8]. It is also important to recognize pauci-immune renal-limited vasculitis as a separate condition. Of note, vasculitis like polyarteritis nodosa (PAN) and giant-cell arteritis (GCA) are not classically associated with ANCA positivity. Lionaki et al. have demonstrated that patients who had kidney-limited vasculitis and glomerulonephritis had MPO positivity in 81% of patients [3]. In the same study, of those patients who had biopsy-proven granulomas instead, 79% had PR3 ANCA positivity, and 21% had MPO ANCA positivity [3]. In our patient, there was rapidly progressive glomerulonephritis and P-ANCA/MPO positivity.

Guillevin et al. in their study demonstrated that 74.5% of patients with microscopic polyangiitis (MPA) have ANCA positivity [9]. Of those 87% have p-ANCA positivity. The MPO antigen positivity helps distinguish MPA from conditions like GCA and PAN. For distinguishing EGPA and MPA, clinical manifestation becomes more important in the correct identification of the diagnosis, since most of the patients with EGPA (73%) are also MPO positive. As far as distinguishing EGPA and GPA is concerned, even though the antigenic target is different, clinical manifestations should also be considered, since, in a small percentage of GPA patients, P-ANCA or MPO positivity can happen as opposed to the classical C-ANCA/PR3 positivity [10].

When testing for vasculitis, it is important to test for antigenic targets by ELISA along with doing an assay for ANCA with IF. A study by Stone et al. demonstrated that the positive predictive value is almost double (45% vs 88%) if both ELISA and IF are positive, as compared to just IF positivity [4]. It is important to recognize that disease severity and flare-up is associated with a higher likelihood of finding ANCA positivity, which implies that false-positives exist, and more so in quiescent disease conditions [11]. 

Boomsma et al. showed that the positive predictive value of rise in titer is modest, with 57% for IF and 71% for ELISA. On the other side, of the patients with rising titers, only 39% had disease flare-up in six months. Hence rising titers by itself cannot be a sole predictor of disease relapse, and the complete clinical picture should be considered prior to any therapeutic changes. The rising titers, however, can make the physician more vigilant to watch for any emerging relapse of vasculitis [12].

The ANCA positivity in the right clinical setting should always be confirmed with tissue biopsy. This is due to the fact that many of the patients would need potent immunosuppressive therapy with the potential for severe adverse effects. Our patient has a tenuous course during the admission, however, once she was stable, a biopsy was pursued. The biopsy was consistent with active glomerulonephritis. Hagen et al. demonstrated that with rapidly progressive glomerulonephritis and ANCA positivity, as was the case in our patient, the positive predictive value for renal crescentic glomerulonephritis is extremely high (98%) [13]. Conversely, a negative ANCA test in such scenarios has a high negative predictive value of 99% [13]. As mentioned above, the positive predictive value of ANCA is higher with more severe disease presentations. In the same study by Hagen et al., a milder renal disease with positive ANCA has a much lower positive predictive value (close to 50%) [13]. This is where the need for biopsy is of vital importance.

In patients with positive ANCA, retesting should be done on a case-by-case basis. There is some data to support that negative ANCA is correlated with lower disease activity, however, more research is needed on this [14].

There are no clinical reference ranges for ANCA titers to associate it with diseases. In the clinical setting, higher titers typically raise more concern. It has been shown that patients with GPA who have higher ANCA titers have greater utilization of healthcare [15]. In another independent study, it was also shown that in patients with MPO vasculitis, higher ANCA antibody titers were associated with poorer renal function and the need for permanent dialysis [16].

## Conclusions

In summary, ANCA disease is a syndrome that can have significant clinical consequences, making the interpretation of ANCA testing critically important. Patterns and titer of ANCA may not correlate clinically with severity, or even presence, of disease, thereby necessitating follow-up and evaluation for clinical context. Given the possibility of rapid deterioration, vigilance and frequent follow-up should be considered in any patient found to have ANCA antibodies.
